# Guided Bilateral Transcanine Implant Placement and Implant-Supported Oral Rehabilitation in a Patient with Progressive Systemic Scleroderma

**DOI:** 10.1155/2021/5576595

**Published:** 2021-07-13

**Authors:** Igor Smojver, Ivan Katalinić, Marko Vuletić, Luka Stojić, Dražena Gerbl, Dragana Gabrić

**Affiliations:** ^1^St. Catherine Specialty Hospital, Zagreb, Croatia; ^2^Department of Oral Surgery, School of Dental Medicine, University of Zagreb, Croatia; ^3^Private Dental Clinic, Zagreb, Croatia; ^4^Clinic of Anesthesiology, University Hospital Clinic Zagreb, Croatia

## Abstract

When faced with a situation where an impacted tooth is in the way of a planned implant, one approach to avoid an invasive surgical procedure and potential associated complications is to place a transcanine implant. The aim of this report was to add a new case of a transimpacted tooth dental implant placement to the existing international literature and to share our experience of transcanine implantation in the maxilla followed by implant prosthodontic rehabilitation of a patient with progressive systemic scleroderma. A 55-year-old woman attended our office for oral cavity assessment and treatment planning for complete oral rehabilitation. Digital planning software was used, and implants were positioned according to a surgical template in regions 13, 16, 23, and 26 (Straumann, Basel, Switzerland) with screw-retained metal-ceramic bridges. Placement of the dental implants through impacted canines and the creation of interfaces other than implant-bone interfaces did not lead to postoperative pain or implant failure. Clinically, overall healing was observed, and the implants were successfully used for implant-supported prosthodontic rehabilitation of the jaw. Within the limitations of this case report, transcanine implantation could represent a valuable alternative to standard implant protocols.

## 1. Introduction

Dental implant placement and implant-supported rehabilitation has become a widely used method for the replacement of missing teeth. Dental implants are usually placed into an edentulous jawbone but only if the three-dimensional volume is sufficient [1]. This can be a challenging task if the bone volume is insufficient or if there is an object obstructing the trajectory of the future implant, like an impacted tooth. The third mandibular molars are the most commonly impacted teeth, followed by the maxillary canines. The rate of appearance of impacted canines is 1–3% in the maxilla [2] and 0.07–1.3% in the mandible [3]. One of the fundamental implantology postulates states that the “implant surface should come into contact only with the bone” [4]. Following this rule, in a situation where an impacted tooth is in the way of a planned implant, a rather invasive surgery to remove the impacted tooth is indicated prior to implant placement. In order to avoid an invasive surgical procedure and the potential complications that may occur following such an extensive surgery, including postoperative pain, swelling, and a large residual bone defect, a staged approach to implant placement requiring two separate surgeries (the impacted tooth is firstly extracted with or without bone augmentation, then the implant is placed later) was first proposed by Davarpanah and Szmukler-Moncler in 2009 [5, 6]. These authors have conducted and published multiple successful cases of transcanine implant placement. Since then, more cases have been recorded [7, 8]; however, the total number of cases is still low as only a small number of patients are suitable for such treatment [9]. For the same reason, long-term follow-up data are also lacking [9]. Scleroderma is an autoimmune multisystem rheumatic condition that affects connective tissues. Oral and facial clinical findings are a mask-like face, thin vermilion border, radial perioral furrows, microstomia, sclerosis of the tongue-tie, and induration of the tongue. Hyposalivation, microstomia, ankyloglossia, limited mouth opening, but also minor manual skills interfere with oral hygiene ability [10, 11]. Due to these facts, the scleroderma condition represents a certain challenge for dental implant placement and/or prosthodontic rehabilitation, as the oral manifestations of scleroderma are directly relevant to the dental treatment plan and long-term management of these patients [10]. Thus, the aim of this report was to add a new case of transimpacted tooth dental implant placement to the existing international literature and to share our experience of transcanine implantation in the maxilla followed by implant prosthodontic rehabilitation of a patient with progressive systemic scleroderma.

## 2. Case Report

**Figure 1:**
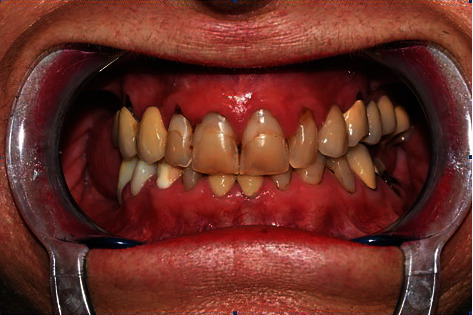
Preoperative intraoral status.

**Figure 2:**
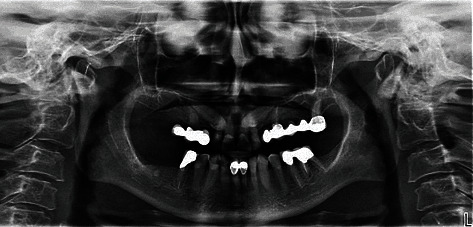
Preoperative panoramic image.

A 55-year-old woman attended our office for oral cavity assessment and treatment planning for complete oral rehabilitation. Her medical records showed she had been diagnosed with progressive systemic sclerosis but had not been taking any medications. She complained of occasional dry mouth and inflamed gums, which is a typical oral manifestation of this disease (Figure 1). An X-ray of the jaw (digital panoramic image) revealed that teeth 14, 24, and 27 needed to be extracted or undergo root canal treatment (Figure 2). Horizontally positioned impacted upper canines were also observed, which were asymptomatic and free from any pathology. Almost all teeth had large, inadequate fillings. After thorough dental examination, an endorestorative treatment was proposed, followed by final rehabilitation of the lower jaw with full ceramic crowns. For the upper jaw, given that the impacted canines would interfere with the traditional implant-supported rehabilitation (fixed ceramic bridges), a combined prosthodontic solution was proposed (fixed ceramic crowns in the frontal region and a removable prosthesis in the back) following all necessary extractions and restorative preparations. The patient was informed that this solution for the upper jaw bares some long-term risks due to her primary diagnosis and chronically dry oral mucosa. The patient refused the removable prosthodontic solution for the upper jaw and asked for a fixed one. Two alternative options were proposed: (1) initial extraction of the impacted canines after bone augmentation, followed by later insertion of the implants, or (2) a novel, experimental approach called transdental implant insertion. The patient was informed that the second procedure was relatively new, had not been fully validated by evidence-based contemporary dentistry, and was based on several published case reports and follow-ups.

Both the benefits (avoidance of aggressive, invasive surgery including bone grafting; reduced financial cost; and increased speed of the final rehabilitation) and risks (prolonged postoperative pain due to canine pulpal tissue damage, followed by removal of the implant and the canine) of this approach were explained to the patient. In the case of failure of the experimental procedure, the conventional treatment (first proposed alternative treatment) would be provided at no additional cost for the patient. The patient chose the transcanine implantation procedure and signed the standardised dental informed consent document along with the general data protection regulation (GDPR) document.

### 2.1. Digital Planning Using coDiagnostiX® Software

**Figure 3:**
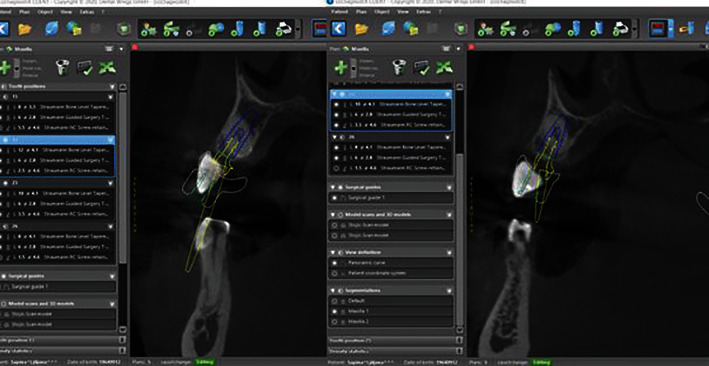
3D planning—transcanine approach in regions 13 and 23.

**Figure 4:**
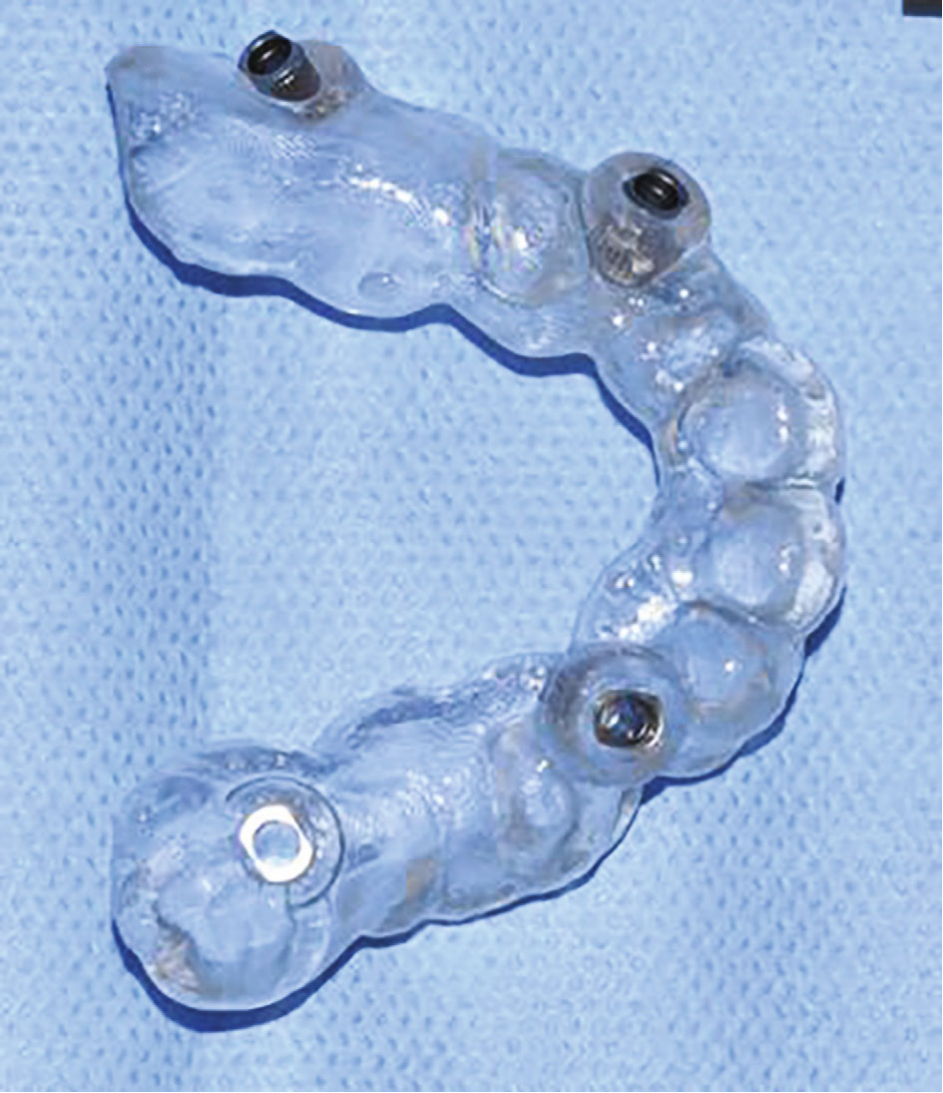
Surgical guide.

In order to properly prepare for the implant placement procedure, coDiagnostiX® (Dental Wings Inc., Canada) digital planning software was used. Silicone impressions (ExaFast Putty and ExaFast NDS, GC Europe, Belgium) of the patient's jaws were sent to the lab, and analog models were cast out of dental stone (BriegelRock Spezial, Briegeldental, Germany). The models were scanned with an E2 lab scanner (3Shape, Copenhagen, Denmark), and a Standard Tessellation Language (STL) file of the patient's upper jaw was created. A CBCT (Cone-Beam Computed Tomography) scan (Orthophos SL, Dentsply Sirona, Bensheim, Germany) of the upper jaw in Digital Imaging and Communications in Medicine (DICOM) format was also taken and imported into the software along with the STL file of the jaw. When these two files were merged, virtual planning could begin. The implant positions were digitally planned considering the final prosthodontic solution (position of the future abutments and crowns) as well as the position of the impacted canines (i.e., avoiding hard canine enamel that could impair the drilling; Figure 3). Water cooling of the implant site was identified as a potential problem, so a surgical guide with only pilot drill guide sleeves was created for positions 13 and 23. The guide was then 3D printed with a Straumann P20+3D printer (Rapid Shape, Heimsheim, Germany) using Pro Surgical Guide clear transparent resin material (P Pro Resin, DeltaMed, Friedberg, Germany; Figure 4).

### 2.2. Surgical Procedure

**Figure 5:**
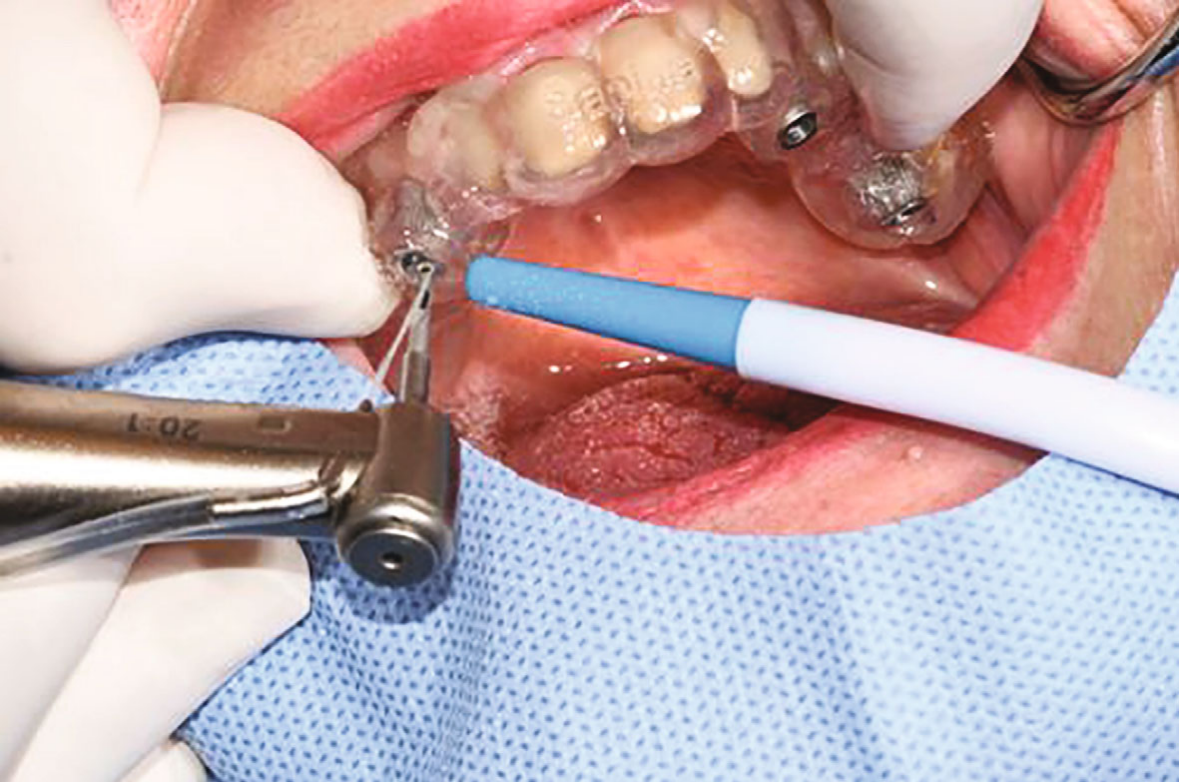
Osteotomy using a pilot drill and a surgical guide.

**Figure 6:**
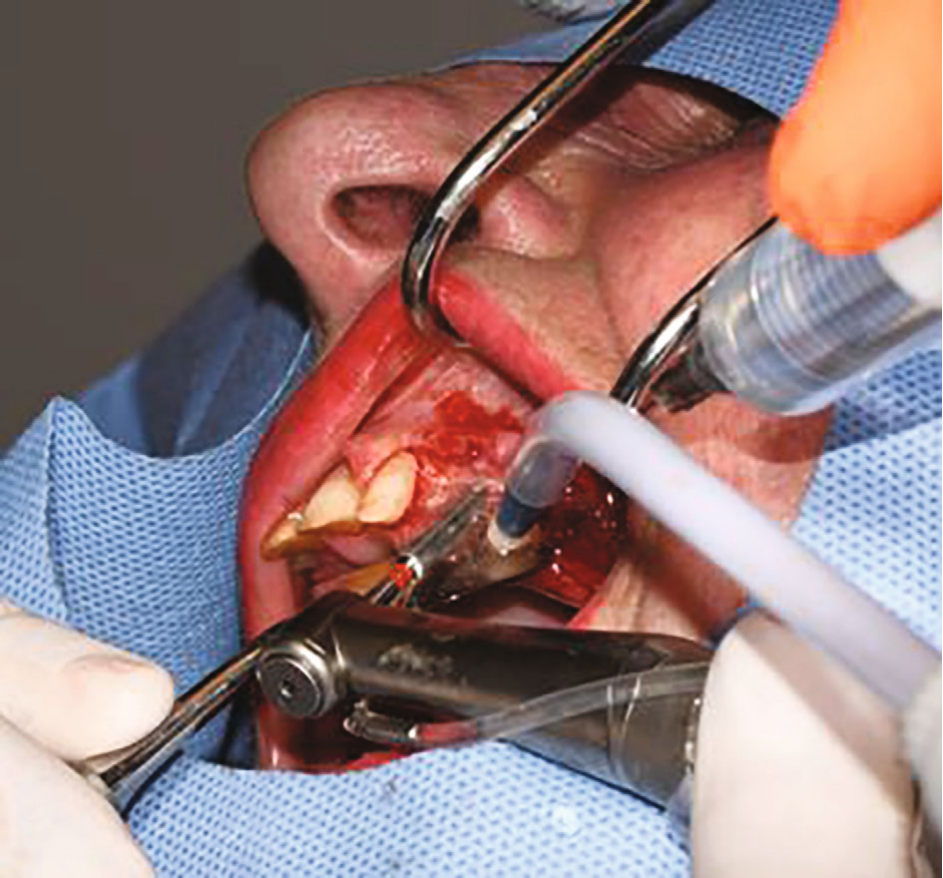
Gradual drilling with additional saline irrigation/cooling.

**Figure 7:**
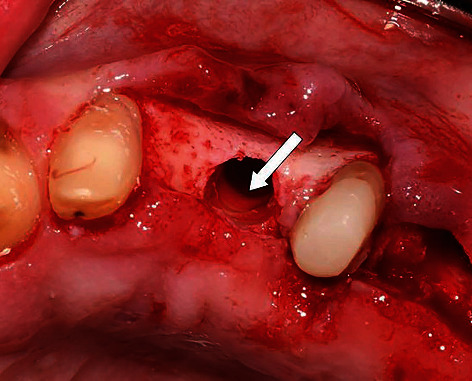
Drill hole through the bone and the impacted tooth (the white arrow).

The greatest concern associated with this guided surgery approach is the potential for excessive heat generation due to friction between the metal sleeves in the surgical template, implant drills, and corresponding guide handles [11–13]. The presence and design of a surgical template may prevent the irrigation fluid from entering the osteotomy site [13, 14]. The excessive heat generated coupled with inadequate irrigation may heat the bone higher than its biological threshold of 47°C and cause irreversible damage. Considering the CBCT measurements of the density of the dentin itself, which in some places exceeded 1000 HU, a protocol for preparation into bone density D1 [15, 16] at the positions of the impacted teeth was prepared. Therefore, we decided to use only a “partial” guided surgical approach for regions 13 and 23 and a drill up to 2.2 mm in diameter (pilot drill) to achieve the planned position of the implants in relation to the axis of the impacted teeth, bearing in mind the need to avoid the tooth enamel (Figures 5–7). A full guided surgery approach was planned at positions 16 and 26.

After raising the entire mucoperiosteal flap and setting the surgical template, osteotomies were performed in regions 16, 13, 23, and 26 with a pilot drill. Classic osteotomy without a surgical template was then done with final drills for implants at positions 13 and 23 (BLT SLActive Roxolid RC 4.1; Institut Straumann AG, Basel, Switzerland) with constant additional irrigation with 0.9% NaCl to ensure adequate cooling (Figure 6).

Implants at positions 16 (BLT SLActive Roxolid NC 3.3; Institut Straumann AG, Basel, Switzerland) and 26 (BLT SLActive Roxolid RC 4.1; Institut Straumann AG, Basel, Switzerland) were placed with a full digital approach without moving the surgical template during the whole procedure. At position 26, a transcrestal sinus lift procedure was performed without the addition of a xenograft.

After the placement of all implants, horizontal augmentation was performed with a xenograft (Xenograft 0.5 g; Institut Straumann AG, Basel, Switzerland) mixed with autologous bone, which was scraped from the nearby buccal plate, and covered with a resorbable collagen membrane (Membrane Flex; Institut Straumann AG, Basel, Switzerland) due to thickening of the residual bone, which, after implant placement, was less than the desired 2 mm buccal bone thickness at positions 16 and 26. Primary wound closure was performed with sutures.

### 2.3. Prosthodontic Procedure

**Figure 8:**
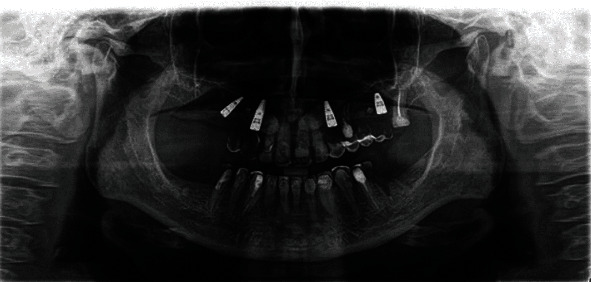
Panoramic image 3 months postop.

**Figure 9:**
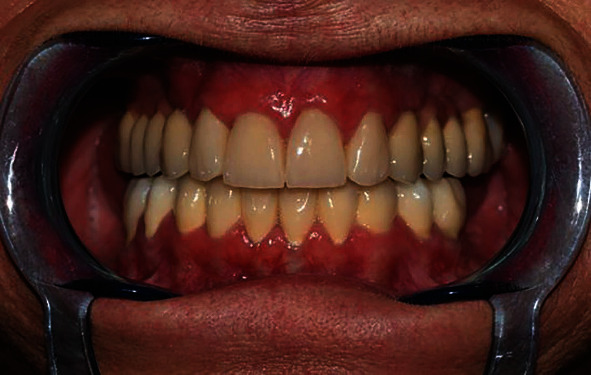
Intraoral status; the definite prosthodontic work.

**Figure 10:**
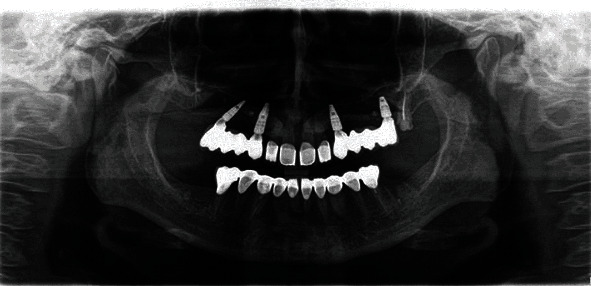
Panoramic image 6 months postop.

**Figure 11:**
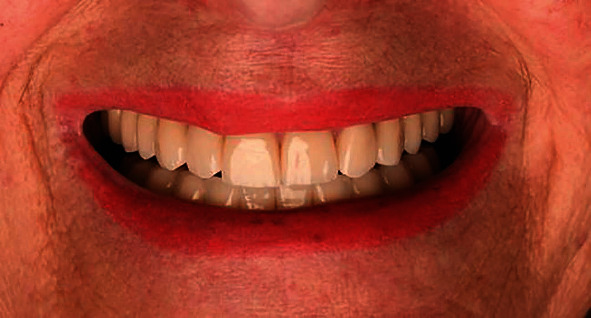
Patient's smile.

During the healing period, the patient was provided with temporary PMMA bridges and crowns which were cemented on the remaining teeth. After 3 months of healing, screw-retained abutments, which were already planned for the surgery itself, were placed and tightened to 35 N/cm according to the manufacturer's recommendation. After preparation of the remaining teeth in both jaws, impressions were taken with an individual tray using a polyether-based material (Impregum Penta Soft, 3M ESPE, Minnesota, USA). Artex Facebow was used to determine and translate intermaxillary relations to the Artex CR articulator (Amann Girrbach AG, Koblach, Austria). Zirconia ceramic crowns (Zolid Zirconia Block, Amann Girrbach, Koblach, Austria; Celtra Ceram Cladding Ceramics, Dentsply, Charlotte, USA) were produced to restore the remaining teeth. The implant crowns were made of a metal base (BEGO™ Chrome Cobalt Implant Bar, Bremen, Germany) coated with ceramics (Duceram Kiss, Dentsply, Charlotte, USA). Definite ceramic crowns were cemented using Fuji Plus cementum (GC Corp., Tokyo, Japan) on the natural teeth, and the implant crowns were tightened to SR abutments with 15 N/cm, as recommended by the manufacturer. Follow-up orthopantomogram was carried out at 3 and 6 months postoperatively (Figures 8–11). The patient was followed up for one year until now after the prosthodontic procedure was done.

## 3. Discussion

When an impacted tooth is in the way of an implant site, following standard implantology protocols, clinicians usually extract the tooth before placing the implant (simultaneous or delayed implantation) [17–20]. Thus, implantation in or through an impacted tooth represents a paradigm shift in the field of dental implantology. Hurzeler et al. [21] made a similar paradigm shift with the so-called “socket shield” technique, where part of the root is deliberately left inside the implant zone to support the buccal bone and prevent complications linked to postextraction bone loss. In the “socket shield” approach, the implant surface comes into contact with the residual root. Histological and microscopic studies have revealed that the threads are partially filled with amorphous mineralised tissue and connective tissue. At a higher magnification, the implant threads were found to have integrated into the newly formed cementum interposed between dentin and the implant, without any fibrous tissues interposed [22]. Another unusual approach to implant placement is when the extracted tooth root is used as a bone “volume booster” in cases with an insufficient alveolar ridge volume. In this procedure, the tooth is extracted, adequately processed, and fixed laterally to the insufficient ridge in order to augment its volume. During implantation, after the healing period, the implant comes into contact with the ankylosed tooth structure, similar to the “socket shield” technique. Researchers have also confirmed the success of this approach [23].

In contrast to the standard implant-tissue interface, transcanine implantation generates four “new” interfaces: (1) an implant-periodontal ligament interface, (2) an implant-cement interface, (3) an implant-dentine interface, and (4) an implant-pulp interface. We tried to avoid implant-enamel contact by using guided implant surgery and digital planning. In theory, the enamel could potentially complicate the implantation process due to its hardness, although Davarpanah et al. [9] stated that this implant-enamel contact did not jeopardize the implantation procedure or implant prognosis.

Our greatest concern was the contact between the pulp and the implant due to the potential for postoperative pain caused by pulpal damage and necrosis. However, no unusual postoperative pain was reported. A similar absence of pain was reported when coronectomy of the wisdom teeth close to the alveolar nerve was performed to avoid injuring the nerve or to move the impacted tooth away from the nerve [24, 25]. This technique was first described by Knutsson et al. [26] in 1989 and has been successfully used since then.

Results similar to ours have been reported in studies conducted by Davarpanah et al. [9] and Haddad et al. [27] in terms of an absence of postoperative pain and complications and final treatment success.

Unfortunately, we were unable to find any other studies, follow-ups or case reports, on this novel approach in the available literature. This is probably because situations where the insertion of an implant is indicated but impacted canines are in the way of the implant site are rare.

Guidelines on various implant treatments of patients with scleroderma or other autoimmune multisystem rheumatic diseases are hard to find in the available literature. Generally, in patients affected by autoimmune multisystem rheumatic diseases like scleroderma, large contact areas between oral mucosa and artificial surfaces of prosthodontic devices (especially removable ones) are less desirable, so a reduction of contact areas between prostheses and mucosa is advised [11]. Dental implants and implant-supported restorations could help achieve such requirements. Results of existing publications demonstrate encouraging outcomes regarding dental implant survival, which are comparable to those of healthy patients [11]. This is in accordance with findings of the current case report; our limited postoperative follow-up period showed no negative effects of dental implant placement on the patient suffering from scleroderma condition, both regarding oral soft tissue health and overall quality of masticatory function.

## 4. Conclusion

Placing dental implants through impacted canines and generating interfaces other than implant-bone interfaces did not lead to postoperative pain or implant failure. Clinically, overall healing was observed, and the implants were successfully used for implant-supported prosthodontic rehabilitation of the jaw. Within the limitations of this case report, transcanine implantation could represent a valuable alternative to standard implantation protocols.

## Data Availability

The data presented in this study are available on request from the corresponding author.
